# Poisonous India Rubber

**Published:** 1870-07

**Authors:** 


### POISONOUS INDIA RUBBER.

White India Rubber, as used in nursing-bottles for infants, contains an active poisonous ingredient, causing sore mouth, eruption, and even death in some cases; its sale is, on this account, prohibited by law in some European countries.

Toys of this material should be carefully kept from young children, whose instinct prompts them to put everything in the mouth which is handed them.

